# Early detection of active Human CytomegaloVirus (hCMV) infection in pregnant women using data generated for noninvasive fetal aneuploidy testing

**DOI:** 10.1016/j.ebiom.2024.104983

**Published:** 2024-02-02

**Authors:** Brigitte H.W. Faas, Galuh Astuti, Willem J.G. Melchers, Annette Reuss, Christian Gilissen, Merryn V.E. Macville, Stijn A.I. Ghesquiere, Leonieke M.H. Houben, Malgorzata Ilona Srebniak, Geert Geeven, Janette C. Rahamat-Langendoen, Erik A. Sistermans, Jasper Linthorst

**Affiliations:** a Department of Human Genetics, Radboud University Medical Center Nijmegen, the Netherlands; b Department of Medical Microbiology, Radboud University Medical Center Nijmegen, the Netherlands; c Department of Obstetrics and Gynecology, Radboud University Medical Center Nijmegen, the Netherlands; d Department of Clinical Genetics, GROW School of Oncology and Reproduction, Maastricht University Medical Center+, the Netherlands; e Department of Clinical Genetics, Erasmus Medical Center Rotterdam, the Netherlands; f Department of Human Genetics, Amsterdam UMC, Location Vrije Universiteit Amsterdam, Amsterdam, the Netherlands; g Amsterdam Reproduction & Development Research Institute, Amsterdam UMC, the Netherlands

**Keywords:** Human cytomegalovirus, hCMV, Screening, Cell-free DNA, Noninvasive prenatal testing

## Abstract

**Background:**

Prenatal hCMV infections can lead to severe embryopathy and neurological sequelae in neonates. Screening during pregnancy is not recommended by global societies, as there is no effective therapy. Recently, several groups showed that maternal–fetal hCMV transmission can be strongly reduced by administering anti-viral agents early in pregnancy. This calls for a screening method to identify at risk pregnancies at an appropriate gestational age, with the possibility for large-scale enrolment. Non-Invasive Prenatal Testing (NIPT) for fetal aneuploidy screening early in pregnancy is already implemented in many countries and performed on a large-scale basis. We investigated the use of whole genome cell-free DNA (cfDNA) sequencing data, generated for the purpose of NIPT, as (pre-)screening tool to identify women with active hCMV-infections, eligible for therapy.

**Methods:**

Coded raw sequencing NIPT data from 204,818 pregnant women from three testing laboratories were analyzed for the presence of hCMV-cfDNA. Samples were stratified by cfDNA-hCMV load. For validation and interpretation, diagnostic hCMV-qPCR and serology testing were performed on a subset of cfDNA-hCMV-positive (n = 112) and -negative (n = 127) samples.

**Findings:**

In 1930 samples (0.94%) hCMV fragments were detected. Validation by hCMV-qPCR showed that samples with high cfDNA-hCMV load tested positive and cfDNA-hCMV-negative samples tested negative. In 32/112 cfDNA-hCMV-positive samples (28.6%) the serological profile suggested a recent primary infection: this was more likely in samples with high cfDNA-hCMV load (78.6%) than in samples with low cfDNA-hCMV load (11.0%). In none of the cfDNA-hCMV-negative samples serology was indicative of a recent primary infection.

**Interpretation:**

Our study shows that large-scale (pre-)screening for both genetic fetal aberrations and active maternal hCMV infections during pregnancy can be combined in one cfDNA sequencing test, performed on a single blood sample, drawn in the first trimester of pregnancy.

**Funding:**

This work was partly funded by the Prenatal Screening Foundation Nijmegen, the Netherlands.

**Research in context**

**Evidence before this study**

We searched PubMed for all published studies between Jan 1, 2018, and Jan 1, 2023 dealing with the use of the presence of Human Cytomegalovirus (hCMV) DNA in circulating cell-free DNA (cfDNA) in the plasma of pregnant women for large-scale screening purposes. We used the search terms “cytomegalovirus cell-free”, “viral DNA pregnancy”, “viral prenatal non-invasive” and “virome pregnant”. No language restrictions were applied. Our search identified four relevant papers published during this timeframe. All four papers showed the technical possibilities to use data generated by whole genome sequencing of cell-free DNA in the plasma of pregnant women for the detection of viral DNA, but validation and interpretation of the data by gold standard molecular and serology methods was not included in the four studies.

**Added value of this study**

In our study, we used coded cfDNA sequencing data from the largest cohort of pregnant women studied so far, nation-wide generated in a clinical setting, to confirm the observations that hCMV-viral-fragments can be detected in cfDNA sequencing data, generated for the purpose of noninvasive genomewide fetal aneuploidy screening. In addition to this confirmation, we performed diagnostic hCMV-qPCR and serology testing on a subset of both cfDNA-hCMV-positive and -negative samples for validation and interpretation. We show that high cfDNA-hCMV-positivity strongly correlates with hCMV-qPCR-positivity and with serology profiles indicative for a recent primary infection (PI) or re-activated past infection. Moreover, PIs were not seen in cfDNA-hCMV-negative samples. To the best of our knowledge, this is the first study that not only deals with the technical possibility to use cfDNA sequencing data generated for the purpose of fetal aneuploidy screening to establish the presence of hCMV-DNA fragments in the plasma of pregnant women, but also includes molecular validation and serology interpretation studies, as a first step towards large-scale maternal (pre-)screening.

**Implications of all the available evidence**

In 2015, an International Congenital Cytomegalovirus Recommendations Group was convened and, in agreement with data in literature and with various national guidelines, advised against Human Cytomegalovirus (hCMV) screening in pregnant women. Therefore, hCMV screening during pregnancy is not recommended in any nation. The most important reason for this is because there are no effective preventive or curative strategies. But recent studies suggest that maternal–fetal transmission after a primary maternal hCMV infection early in pregnancy can be strongly reduced by administering high dose Hyperimmune Globin (HIG) or antiviral agents early in pregnancy. Even though large-scale high-quality studies to validate these observations are still needed, these developments call for a screening method to identify pregnant women at risk at an appropriate gestational age, with the possibility for large-scale enrolment. The data of our study suggest that hCMV-screening using cfDNA sequencing data generated for the purpose of fetal aneuploidy screening could become a first step for the timely identification of active hCMV infections in pregnant women. As such, the data of our study, should be taken into account when, in the light of possible therapeutic agents, re-evaluating the current guidelines dealing with hCMV screening during pregnancy.

## Introduction

Human CytoMegaloVirus (hCMV, Human Herpesvirus 5) infection is the most common congenital viral infection worldwide, with a birth prevalence of 0.5%–1% in the United States and Europe.^1^ Even though in adults the course of an infection is often asymptomatic, fetal infection, resulting from maternal–fetal transmission, can lead to severe embryopathy, even with intrauterine fetal death. In neonates, in utero exposure to hCMV can result in hearing loss and severe neurological problems.^2^ As recently reconfirmed by Chatzakis et al.*,*^3^ the maternal–fetal transmission rate increases as the onset of the maternal infection occurs at a later gestational age, from 36.8% with onset in the first trimester, to 66.2% in the third trimester. However, severe phenotypes are only seen if maternal infection has occurred periconceptional or during the first trimester. This is mainly based on observations in pregnant women with primary infections (PIs), but congenital hCMV infections can also result from maternal nonprimary infections (NPI), i.e. re-activated past infections or reinfections.^4^

Up to now, even though congenital hCMV infection is considered a major health concern, (inter)national societies do not recommend screening for hCMV during pregnancy, as the clinical severity of a fetal infection can vary largely and there is no (inter)nationally accepted therapy yet. Previously, several authors studied the effectiveness of maternally administering Hyperimmune Globin (HIG) for the reduction of maternal–fetal transmission after a maternal PI in randomized, placebo-controlled trials, with disappointing results.^5^, ^6^, ^7^, ^8^ Recently, however, data from uncontrolled/observational studies from another group^9^^,^^10^ suggest that reduction in transmission might be achieved by administration of higher HIG dosages, employing shorter intervals between treatments, and targeting therapy to earlier gestational ages at the time of primary infection with shorter intervals from seroconversion to treatment initiation. Moreover, studies on administering Valaciclovir, a well-known antiviral drug, also showed a reduction in maternal–fetal transmission,^11^^,^^12^ when preferably starting treatment before 14 weeks of gestation. Furthermore, Roberts et al.^13^ described two pregnant women, in whom maternal–fetal transmission had already occurred, who were treated with antivirals and whose fetuses did not develop severe embryopathy. Even though large-scale high-quality studies are needed to validate these data and to more accurately define the group of pregnant women who would benefit most from such interventions, these developments call for a screening method to identify pregnancies at risk at an appropriate gestational age, with the possibility for large-scale enrolment. So far, serological testing, with additional molecular testing in specific cases, is the routine hCMV diagnostic strategy. However, with serological methods only recent PIs can be identified: NPIs, that can also cause severe embryopathy, cannot be distinguished from harmless latent past infections.

Non-Invasive Prenatal Testing (NIPT) for the detection of fetal aneuploidies is offered in many countries to all pregnant women, early in pregnancy. It is offered from week 10–11 of pregnancy and can involve shallow whole genome sequencing of double-stranded cell-free DNA (cfDNA) fragments from maternal plasma. As hCMV is a double-stranded DNA virus, hCMV fragments in the maternal plasma are sequenced as well. Previously, several authors demonstrated that hCMV-fragments can be detected in the pool of sequenced cfDNA, but its clinical significance has not been determined.^14^, ^15^, ^16^, ^17^, ^18^ hCMV infections are predominantly spread through the body via cell-to-cell interactions and clinically relevant hCMV viraemia is mostly cell-associated.^19^ As the highly fragmented genomes present in the plasma are considered non-infectious, a test determining the presence of hCMV fragments in plasma is to be regarded as a test that determines the presence or absence of a biomarker. But if the detection of hCMV-cfDNA in an NIPT sample reflects the hCMV status of a pregnant woman, this would open the way to a relatively low-cost combined large-scale screening test. Recently, the American College of Medical Genetics and Genomics (ACMG) strongly recommended NIPT over traditional screening methods for all pregnant women.^20^ In The Netherlands, NIPT has been offered to all pregnant women as a first tier test since 2017 from the 11th week of pregnancy, with a mean turn-around time (TAT) of 6.5 business days.^21^ This means most of the NIPT results are available well before 14 weeks of gestation and thus information on the hCMV status of the pregnant woman, if obtained from the same data, will be reported in time to potentially start administering therapeutic agents. To further investigate the potential use of cfDNA sequencing data for large-scale hCMV screening in pregnant women to identify pregnancies at risk for maternal–fetal transmission, we performed a study on both the presence and the interpretation of hCMV fragments in the cfDNA-sequencing data of a large cohort of pregnant women, obtained in a clinical setting.

## Methods

### Study design

To retrospectively study the presence of cfDNA-hCMV-fragments, a consecutive coded dataset of whole genome cfDNA sequencing data, generated between June 2018 and May 2021, was collected from the three NIPT centers in the Netherlands (Maastricht University Medical Center, Maastricht; Amsterdam University Medical Center, Amsterdam; Erasmus Medical Center, Rotterdam). The data were generated as part of the TRIDENT-2 study, a nationwide study on the implementation of Non-Invasive Prenatal Testing (NIPT) in The Netherlands, in which NIPT was offered to all pregnant women as a first-tier screening test between April 2017 and 2023.^21^ Study population characteristics were comparable to those of the first-year TRIDENT-2 cohort studied by van der Meij et al.*,*^21^ with a mean gestational age of 11.9 weeks at the time of blood draw.

After excluding data from samples that failed to meet the national quality criteria for regular NIPT, including a minimal number of sequenced reads of 10 million, the dataset under study contained data from 204,818 pregnant women. After bioinformatic analysis of the data, 3–3.5 mL left-over plasma (Streck-tube plasma stored at −80 °C) from a subset of samples was sent to the Department of Medical Microbiology at the Radboud university medical center, Nijmegen (The Netherlands), for validation of the data by hCMV-qPCR and interpretation by serological testing, both being the current gold standards in hCMV diagnostics. The cfDNA-hCMV results were not sent with the plasma samples. Based on serology data, the hCMV-status of the samples were categorized into 1) seronegative, 2) seropositive and indicative for a recent primary infection (PI) or 3) seropositive from a past infection. In case of a serological profile suggestive for a PI, the onset of the infection was estimated by avidity testing. As in this study coded data are used, patient details and files remained unknown to the researchers, and therefore the validation endpoint was a comparison between the results obtained with the cfDNA-hCMV sequencing data and the hCMV diagnostic results.

### cfDNA sequencing data generation

Sequencing methods are as described by van der Meij et al.*,*^21^ with some modifications. Briefly, upon isolation of cfDNA from blood drawn in Cell-Free DNA BCT CE tubes (Streck, La Vista, NE, USA), library preparation was performed using VeriSeq™ NIPT reagents (Illumina, San Diego, CA, USA) and libraries were paired-end sequenced on a NextSeq500 system (Illumina, San Diego, CA, USA). Demultiplexing and adapter trimming of raw data was performed using bcl2fastq (version 2.17).

### cfDNA-hCMV-analysis

Samtools (version 1.6) was used to extract all reads that did not align to the human reference genome (build GRCh37 or GRCh38), which were subsequently aligned to the human herpesvirus 5 strain Merlin genome (Refseq assembly accession GCF_000845245.1) using BWA mem (version 0.7.17). After aligning the nonhuman reads to the viral reference genome and masking low-complexity regions using RepeatMasker (https://github.com/rmhubley/RepeatMasker) or the DUST algorithm (as implemented in the meme-suite20 (version 5.3), with default settings to mask regions of low-complexity sequence) only paired reads, derived from the same viral fragment, were included in the downstream analysis. Sample ID was indicated by read group tags and the fragment number per sample as well as the number of hCMV fragments per million sequenced reads (FPM) were calculated.

### Validation of the cfDNA-hCMV data by hCMV-qPCR

For hCMV-qPCR, nucleic acids were extracted from left-over plasma samples using MagNaPure 96 (Roche Diagnostics, The Netherlands) and subsequent real-time quantitative PCR (qPCR) was performed on the LightCycler 480 using RealStar®CMV assay (Altona Diagnostics, Germany). This assay amplifies a 74-basepairs fragment within the immediate early protein UL122 region. Viral load was determined and categorized as either 1) negative, 2) positive with viral load of <100 IU/mL (very low, non-quantifiable, but detectable load), or 3) positive with X IU/mL (in case viral load was >100 IU/mL).

### Serological interpretation of the cfDNA-hCMV data

hCMV serology testing (anti-hCMV-IgM and anti-hCMV-IgG) was performed using the Liaison® IgM and IgG II assay (DiaSorin, Italy). In case of intermediate or positive anti-hCMV-IgM (defined as ≥18 AU/ml), combined with anti-hCMV-IgG-positivity (≥12 AU/ml), anti-hCMV-IgG-avidity testing was performed (Liaison® CMV IgG Avidity II assay (DiaSorin, Italy)). In accordance with the instructions of the manufacturer, specific anti-hCMV-IgG with low avidity (<0.150) was considered indicative of a PI within the last three months, although some patients will keep low avidity anti-hCMV-IgG for a longer period than 3 months. High avidity (≥0.250) excluded a PI within the last three months, but does not exclude an active infection due to a re-infection or re-activation. As intermediate indexes (>0.150 and < 0.250) can also indicate a PI within the last three months, for the purpose of the current study these were also categorized as recent PIs. Anti-hCMV-IgG-positivity combined with -IgM-negativity was considered indicative for a past infection.

### Statistical methods

Statistical calculations were performed in SciPy, version 1.7.3. Standard deviations (and the derived confidence intervals) of reported sample proportions (or percentages) are calculated by the normal approximation (Wald's method) using the formula: $\sigma_{p}=\sqrt{\;\frac{p\left(1-p\right)}{n}\;}$, where $\sigma_{p}$ is the standard deviation of the proportion p, and where n is the sample size. Standard deviations of the product of two independent proportions were calculated by propagating the errors of the individual proportions, using the formula: $\sigma_{12}=\sqrt{\;{(\sigma }_{1}^{2}+\mu_{1}^{2}){(\sigma }_{2}^{2}+\mu_{2}^{2})\;–\;\left(\mu_{1}^{2}*\mu_{2}^{2}\right)}$, where $\sigma_{12}$ is the standard deviation of the product of proportions $\mu_{1}$ and $\mu_{2}$ and their standard deviations $\sigma_{1}$ and $\sigma_{2}$. To determine the standard deviation of the sum of two independent proportions, the following formula was used $\sigma_{1+2}=\sqrt{\;\sigma_{1}^{2}+\sigma_{2}^{2}}$, where $\sigma_{1+2}$ is the standard deviation of the sum of proportions p1 and p2.

In general, *p* values less than 0.05 were considered statistically significant.

### Ethics

Approval for the TRIDENT-2 study, as part of which the NGS data were generated, was granted by the Dutch Ministry of Health, Welfare, and Sport (license 1017420-153371-PG) and approved by the Medical Ethical Committees of the participating medical centers. Researchers of the current study did not have access to patient details, sequencing data were coded for the researchers and only data from women who gave written informed consent for use of their data for research purposes were included. Results were not communicated with patients. More information about the inclusion and exclusion criteria for the TRIDENT-2 study can be found in a previous publication.^21^

### Role of funding source

The funder of the study had no role in the study design, data collection, data analysis, data interpretation, or writing of the report.

## Results

### cfDNA-hCMV-fragment detection rate and mapping

After sequencing, the mean number of reads per sample was 24.27 × 10^6^ (SD 8.1 × 10^6^), with a median of 23.33 (interquartile range 20.13–27.08 × 10^6^) reads. hCMV fragments were detected in 2349 samples (1.15%). The number of fragments in hCMV-positive samples ranged from 1 to 196, with a mean of 2.96 and a median of 1 fragment per sample (interquartile range 1–2). The mean hCMV fragment length in cfDNA was significantly shorter than that of human-derived cfDNA (111 v. 171 bp, Wilcoxon's rank sum test *p* = 5.4 × 10^−178^). Moreover, in samples with at least 40 viral fragments (n = 49), the fragment length distribution was studied and showed two main viral peaks: one of ∼63 basepairs (bp) and one of ∼150 bp.

Alignment of the cfDNA-hCMV fragments to the viral genome revealed a non-uniform distribution of hCMV fragments across the viral reference genome, as a disproportionate number of fragments mapped to the 175-176-kb region of the viral reference genome. The mean size of these fragments was 127 bp, which is significantly larger than that of the viral fragments (Wilcoxon's rank sum test *p* = 3.757 × 10^−12^), and significantly shorter than that of the human fragments (Wilcoxon's rank sum test *p* = 2.2 × 10^−16^). Even though in our analysis only reads that did not map to the human genome were included and the fragment length of these fragments did not match the fragment length of the human cfDNA fragments, we tried to re-map the fragments to the human genome to confirm these fragments did not originate from the human genome. Indeed, none of these fragments could be mapped to the most recent human genome assembly (T2T-CHM13v2.0). In total, in 419 cfDNA-hCMV-positive samples this fragment was detected exclusively (307 with one fragment and 112 with multiple fragments). Of all samples with fragments mapping to the 175-176-kb region, 56.8% originated from one center, mostly investigated within the same month. Table 1 shows the cfDNA-hCMV loads, normalized to fragments per million (FPM) sequenced reads, with both in- and exclusion of fragments mapping to the 175-176-kb region. Overrepresentation of fragments mapping to this region and nonspecific alignment of fragments was previously also noted by others.^14^^,^^16^^,^^22^ Strong et al.^22^ attributed the overrepresentation of such fragments to laboratory plasmid contamination. Even though further research is needed to provide a conclusive answer on the origin of the fragments in this region, for this study we decided to exclude these fragments from the analysis, which resulted in a remainder of 1930 samples with cfDNA-hCMV fragments (0.94%; Table 1). After exclusion, samples with exclusively this fragment were categorized as cfDNA-hCMV-negative.

**Table 1:** Overview of the viral loads (total number of samples n = 204,818).

	Number of samples including fragments mapping to the 175-176-kb region	Percentage	Number of samples excluding fragments mapping to the 175-176-kb region	Percentage
**Total number of positive samples/percentage positive samples in total cohort**	2349	1.1469	1930	0.9423
**Subdivided per FPM**				
0	202,469	98.8531	202,888	99.0577
<0.1	1896	0.9257	1553	0.7582
≥0.1–<0.2	240	0.1172	178	0.0869
≥0.2–<0.3	65	0.0317	55	0.0269
≥0.3	148	0.0723	144	0.0703

FPM = hCMV Fragments Per Million sequenced reads.

### cfDNA-hCMV data validation using hCMV-qPCR

**Fig. 1:**
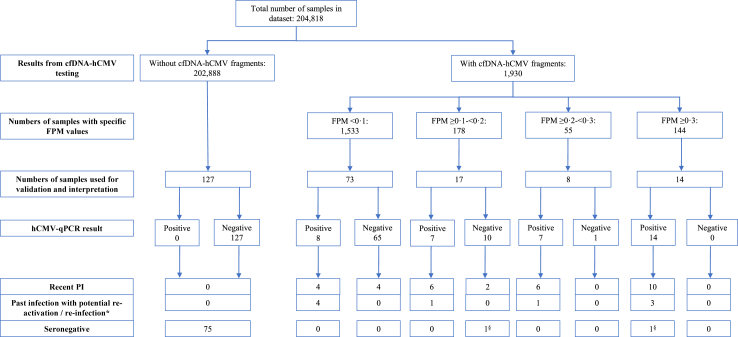
**Flow chart of the study design, including results.** ∗Samples with FPM >0 and positive qPCR ^§^suggestive for a very recent PI, without immune response yet and therefore serologically undetectable (FPM = hCMV Fragments per Million sequenced reads; PI = Primary Infection).

To validate the data, routine hCMV-qPCR was carried out on cfDNA from left-over plasma of 239 samples, selected on the basis of availability of left-over material from a subset of samples: these were 127 cfDNA-hCMV-negative samples and 112 -positive (see Fig. 1).

All cfDNA-hCMV-negative samples were also negative with hCMV-qPCR. This group included six samples with exclusively fragments in the 175-176-kb region, which reinforces the decision to consider these fragments as artefacts. Of the cfDNA-hCMV-positive cases, 67.8% (76/112) tested negative with hCMV-qPCR: these were predominantly samples with an FPM value < 0.1. In samples with FPM ≥0.3, hCMV-qPCR always showed a positive result. There was a positive correlation between increasing mean viral load estimates by qPCR and increasing FPM values (Pearson's correlation *r* = 0.89, *p* = 2.2 × 10^−16^): mean loads were 276 IU/mL (range <100–691) in samples with FPM value < 0.1, 338 IU/mL (range <100–782) in samples with FPM ≥0.1–<0.2, 369 IU/mL (range 158–636) in samples with FPM ≥0.2–<0.3 and 1844 IU/mL (range 187–6590) in samples with FPM ≥0.3.

### cfDNA-hCMV data interpretation using serological testing

As shown in Table 2 and Fig. 1, in none of the cfDNA-hCMV-negative samples diagnostic serology results suggested a recent primary infection (PI), but serological profiles that could fit a recent PI were seen in 32 cfDNA-hCMV-positive plasma samples (28.6%). Overall, samples with higher FPM values (≥0.2) were more likely to be associated with PIs than with past infections (Chi-square test: χ2 = 25.2, *p* = 5.27 x 10^−7^).

**Table 2:** Serology data of samples with and without cfDNA-hCMV-fragments.

FPM	Number of samples	hCMV status, based on serological results						
		Seronegative	Recent PI			Past infection		
			Onset <3 months ago^a^	Intermediate avidity index	% recent PI	IgG-pos IgM-neg	IgG-pos IgM-pos high avidity	% past infection
0	127	75	–	–	–	52	–	40
>0–<0.1	73	–	6	2	11	60	5	89
≥0.1–<0.2	17	1	6	2	47^b^	5	3	47
≥0.2–<0.3	8	–	6	–	75	1	1	25
≥0.3	14	1	10	–	71^b^	3	–	21
**Total of samples with FPM >0**	**112**	**2**	**28**	**4**	**28**^b^	**69**	**9**	**70**

FPM = hCMV Fragments Per Million sequenced reads; PI = Primary Infection.

a Onset <3 months: samples with IgG- and IgM-positivity and avidity index <0.150 and samples with IgG-negativity and IgM-positivity but cfDNA- and hCMV-qPCR-positivity.

b Based on serological data. cfDNA-hCMV-positive but seronegative samples are not included in this percentage.

Of the in total 239 serologically tested samples, 54.4% (n = 130, 52 cfDNA-hCMV-negative and 78 cfDNA-hCMV-positive (Table 2)) showed profiles fitting a past infection, with the highest percentage observed in the cfDNA-hCMV-positive samples with FPM <0.1 (89% (65/73 samples)). Even though low cfDNA-hCMV loads might be present as background in these samples, we hypothesize that samples with cfDNA-hCMV fragments and a positive hCMV-qPCR result are related to re-activation.

Seronegativity was observed in 75 cfDNA-hCMV-negative samples (59%) and two cfDNA-hCMV-positive samples (1.8%).

Of the 76 cfDNA-hCMV-positive samples with a negative qPCR, six samples (7.9%) showed serological profiles that could fit a recent PI.

### Extrapolation to population-based first trimester screening

**Fig. 2:**
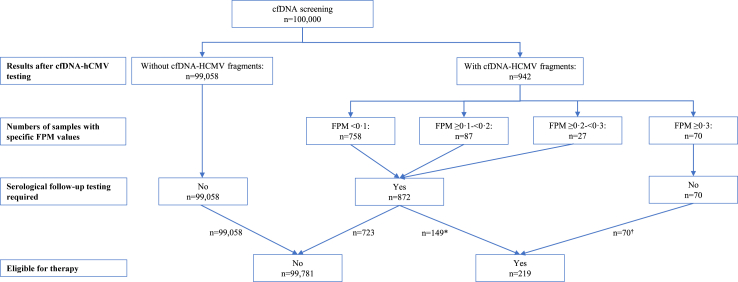
**Possible hCMV screening scenario in a population of 100,000 pregnant women**. ∗Recent PIs only. In this group, potential re-activations/re-infections cannot be identified. ^**†**^Recent PIs (n = 55) and re-activation/re-infections (n = 15), based on FPM ≥0.3 (for 95% CI intervals, see Supplementary Table S2).

Validation and interpretation studies were carried out on left-over materials of a subset of samples from the total cohort. However, due to unavailable left-over material, not all positive samples of the subset could be included. Furthermore, samples with specific FPMs in the entire cohort were not equally represented in the validation cohort (Supplementary Tables S1 and S2). Taking into account the confidence intervals in the different categories, we estimate that in 0.2% (95% CI, 0.14%–0.26%) of (first trimester) pregnancies tested with NIPT a recent PI can be detected by serological screening of all cfDNA-hCMV-positive pregnancies. Extrapolation to a population of e.g. 100,000 pregnant women, suggests that, if NIPT would be implemented for large-scale first trimester (pre-)hCMV-screening, 942 (95% CI, 856–1028) cfDNA-hCMV-positive pregnant women would be identified (∼0.94%), and in 99.06% no cfDNA-hCMV fragments would be detected (Supplementary Table S2 and Fig. 2).

## Discussion

In the largest cohort of pregnant women studied so far, derived from a clinical setting, we confirm previous observations^14^, ^15^, ^16^, ^17^, ^18^ that hCMV-fragments can be detected in cfDNA sequencing data, generated for the purpose of noninvasive genomewide fetal aneuploidy screening. In addition, we show that for cfDNA-hCMV-positive pregnancies, FPM correlates with an active infection, either a recent PI or a re-activated past infection, whereas all cfDNA-hCMV-negative samples are also negative with qPCR, with serology showing no profiles fitting recent PIs. This makes cfDNA-hCMV analysis a valuable tool to detect any active hCMV-infection early in pregnancy (and potentially exclude a large cohort of pregnant women not at risk), with the potential to serve as a large-scale high throughput (pre-)screening method to identify women at risk for maternal–fetal viral transmission at the critical stage for causing severe clinical consequences.

The observed frequency of 0.94% (n = 204,818; 95% CI: 0.90%–0.98%) of cfDNA samples containing hCMV fragments is higher than the frequency of 0.40% (n = 141,431; 95% CI: 0.37%–0.43%), observed by Liu et al. in the Chinese pregnant women population.^17^ This difference might be caused by slightly different detection methods, but may also reflect a true difference between population frequencies, and/or a combination of these.

Extrapolation of our data suggests that with cfDNA-hCMV testing recent PIs are detected in approximately 0.2% (95% CI: 0.14%–0.26%) of the Dutch pregnant population. This is lower than the 0.45% determined by both Seror et al.^23^ and Jin et al.*,*^24^ based on serological data in other populations. More data are needed to better determine the seroconversion rate per population in the first trimester.

Validation of the data by hCMV-qPCR on a subset of samples showed that all cfDNA-hCMV-negative samples were qPCR-negative. In 67.8% of the cfDNA-hCMV-positive samples in the validation set, hCMV-qPCR was also negative, but samples with FPM ≥0.3 always showed a positive result. The qPCR used in this study has a detection limit of 60 IU/mL, which is sufficient for its pretended use but might be insufficient to detect very low numbers of viral fragments. Moreover, the qPCR primers are directed towards specific regions of the hCMV genome, whereas with cfDNA analysis all regions of the genome are taken into account, and cfDNA fragments might be shorter than the qPCR-target fragments.

Analysis of the fragment length distribution revealed two main viral peaks: the reason for the presence of these two peaks is unclear, but might be related to the underlying biological mechanism via which the viral DNA is fragmented and released into the plasma.

In none of the 127 cfDNA-hCMV-negative samples serology suggested a recent infection: 59.1% of the samples was seronegative and 40.9% showed a profile fitting a past infection. This seroprevalence falls within the range of the 36.9% seroprevalence reported in Dutch women of reproductive age.^25^

In 28.6% of the cfDNA-hCMV-positive samples, serology results suggested a recent PI. Two cfDNA-hCMV-positive samples were seronegative. Despite the serology data of these two samples, we categorized these as very recent PIs, with viraemia, but without immune response yet, and therefore too early to be detected with serology. This brings the total of recent PIs to 34, which is 30.4% of the cfDNA-hCMV-positive samples. The positive predictive value for detecting a recent PI correlates with FPM, as in samples with FPMs ≥0.2–<0.3 and ≥ 0.3 this was 75% and 78.6%, respectively, whereas in samples with FPM <0.1 this was 10.9%. Serologically, three of the 14 samples with FPM ≥0.3 showed profiles fitting past infections. The viral loads of these three samples (187, 1650 and 6590 IU/mL) are suggestive for an active infection, being either a re-activation or reinfection. This suggests that by high FPM, e.g. FPM ≥0.3, any active infection can be identified in maternal blood. This is in contrast to serology testing, by which only recent PIs can be identified. In the current study, most of the cfDNA-hCMV-positive samples with serology profiles fitting a past infection had low FPM-values. We assume that those with a positive hCMV-qPCR result are related to re-activated infections or reinfections, but this cannot be concluded with certainty, as a low background level of viral fragments might exist in seropositive pregnant women.

In a prenatal diagnostic setting, ultrasound anomalies resulting from fetal hCMV-infections are often detected during the 20-weeks ultrasound scan. Upon such a finding it is relevant to determine whether an active infection has occurred during the first trimester or periconceptionally, as only those infections can explain fetal ultrasound anomalies. Serology testing is currently the gold standard for diagnostic hCMV-testing, as with serology testing a recent PI can still be established when viraemia, which appears from week 3–6 after contact for a median of 6 weeks,^26^ might not be detectable anymore. However, future screening in the first trimester should be focused on the detection of currently active infections, which does make (pre-)screening for the presence of viral DNA instead of screening by serological methods a reasonable option. Our study shows that by using the data generated for whole genome NIPT, in a minority of women, in whom cfDNA-hCMV-fragments are detected, follow-up serological testing might be indicated, whereas in the vast majority of women, in whom no cfDNA-hCMV fragments are detected, it is not. As high FPM-values are highly suggestive for any active infection, a possible scenario would be that women with FPM ≥0.3 can directly opt for therapy without follow-up serology testing, assuming therapy not only reduces maternal–fetal transmission in case of a recent PI, but in any active infection. The clinical relevance of low FPM values is uncertain, but as a low FPM value might also be related to a very early (upcoming) viraemia, also in women with FPM <0.3 serology testing will be indicated. Additional studies will be needed to determine whether women with different FPM values should be treated with similar or different dosages of therapeutic agents. One should keep in mind that the detection of cfDNA-hCMV fragments in plasma does not automatically imply maternal–fetal transmission, but might serve as a marker to identify women at risk.

By extrapolating our data to a population of 100,000 pregnant women, we calculated that with 95% certainty, cfDNA-hCMV would be detected in between 875 and 1028 pregnant women. We also estimate that, with 95% certainty, serological testing of these women would identify between 142 and 267 recent PIs. In addition to this, between 9 and 21 women with FPM ≥0.3 and a serological profile fitting a past infection would be identified, most likely related to an active reactivated infection (Fig. 2). One should, however, bear in mind that, just as with serology testing, re-activated infections with FPM <0.3 will remain undetected. Furthermore, these numbers are based on the Dutch population and might be different in other populations.

An important strength of our study is that the cfDNA-hCMV data of a subset of samples is validated by routine microbiological diagnostic methods. Moreover, by including all eligible data of the three NIPT laboratories in The Netherlands we created the largest clinical dataset studied so far, generated with the same technology and within the same timeframe.

A major limitation is that the study was carried out on coded data and the researchers did not have access to patient details and medical records. Therefore, the clinical relevance of the active infections could not be established by looking at clinical data, only by comparison with the current gold standard. Furthermore, we could not take into account any possible confounders that might influence the detection of cfDNA-hCMV, such as maternal age, gestational age or BMI.^16^ Another drawback is the relatively limited number of samples that could be included in the validation and interpretation studies. Even though based on our data a large number of active infections in the cfDNA-hCMV-negative group is not likely, the number of cfDNA-hCMV-negative samples included in the validation set is too low to draw firm conclusions on the absence of active infections in these samples.

In conclusion, we show that large scale screening for both genetic fetal aberrations and active maternal hCMV-infections during pregnancy can be combined in one test, performed on a single blood sample, drawn in the first trimester of pregnancy. As NIPT is offered from week 10–11 of pregnancy and the results of both the genetic and the viral screening can easily be issued within two weeks after sampling, women will still be able to opt for the administration of therapeutic agents to prevent maternal–fetal transmission. The current study only dealt with the feasibility of hCMV-screening using cfDNA-sequencing data that have been generated for the purpose of genome-wide fetal aneuploidy screening, to identify pregnant women with an active hCMV-infection. It shows very promising results, but also calls for future work to evaluate the clinical relevance of these findings and potential implementation strategies.

## Contributors

BHWF designed the project and received the funding. GA (funded by the SPN), JL, SAIG, GG, LMHH, AR, CG, EAS, MVEM, and MIS selected data or materials used for the analyses. GA, GG and JL performed the bio-informatic analyses. WJGM performed the studies on the validation samples. BHWF, EAS, JL, GA, JCRL, AR, CG, MVEM and MIS conducted critical data analysis and interpretations. BHWF, GA and JL accessed and verified the underlying data. BHWF wrote the initial draft, subsequent revisions, and final version of the paper with support from all co-authors. All authors critically reviewed the paper and provided crucial intellectual content. All authors approved the final version and agreed to be accountable for all aspects of the work.

## Data sharing statement

The data used in this study are from women participating in the TRIDENT-2 study, who consented for use of their coded data for research purposes. All the data used in this study are routinely collected and contain no patient-specific information. FASTQ, BAM and VCFs cannot be disclosed. The anonymized viral dataset is available upon reasonable request to the corresponding author, subject to the agreement of all co-authors.

## Declaration of interests

EAS declares to have received a grant for research with focus on the heel prick. He is a board member of the Dutch Society for Laboratory specialists clinical genetics and of the Genomics Quality Assessment consortium (GenQA), and received support for attending conferences from the latter. The other authors declare no conflicts of interest.
