# Supplementary material for: Early detection of active Human CytomegaloVirus (hCMV) infection in pregnant women using data generated for noninvasive fetal aneuploidy testing
Source: eBioMedicine. 2024 Feb 2;100:104983. doi: 10.1016/j.ebiom.2024.104983 (PMC10878988; doi:10.1016/j.ebiom.2024.104983)
Supplement: Supplementary Tables [file mmc1.docx]

**Faas *et al*.:**

**Early detection of active Human CytomegaloVirus (hCMV) infection in pregnant women using data generated for noninvasive fetal aneuploidy testing.**

**Supplementary Tables:**

**Supplementary Table 1: Representation of the cfDNA-hCMV-positive samples with different FPMs in the entire cohort and in the validation/interpretation cohort**

|  | **Number of positive samples in entire cohort** | **% of positive samples in entire cohort** | **Number of positive samples in validation cohort** | **% of positive samples in validation cohort** |
| --- | --- | --- | --- | --- |
| **Total** | 1,930 | 100 | 112 | 100 |
| **Subdivided per FPM** |  |  |  |  |
| <0·1 | 1,553 | 80·5 | 73 | 65·2 |
| ≥0·1-<0·2 | 178 | 9·2 | 17 | 15·2 |
| ≥0·2-<0·3 | 55 | 2·8 | 8 | 7·1 |
| ≥0·3 | 144 | 7·5 | 14 | 12·5 |

FPM = hCMV Fragments Per Million sequenced reads

**Supplementary Table 2: Extrapolation of the data of the current study to a 100,000 pregnant population.**

| **FPM** | **Detection rate in current study population (%)** | **Validation cohort** | **Extrapolation to a 100,000 pregnant population** | |
| --- | --- | --- | --- | --- |
|  |  |  |  |  |
|  | **Percentage**  **(SD)** | Percentage recent PIs  (95% CI) ^§^ | Expected numbers of samples with fragments  (95% CI) | Expected number of PIs  (95% CI) |
| >0-<0·1 | 0·7582  (1·92 x 10^-4^) | 11·0  (3·79 to 18·1) | 758  (681 to 835) | 83  (29 to 138) |
| ≥0·1-<0·2 | 0·0869  (6·51 x 10^-5^) | 52·9  (29·2 to 76·7) | 87  (61 to 113) | 46  (24 to 68) |
| ≥0·2-<0.3 | 0·0269  (3·62 x 10^-5^) | 75·0  (44·9 to 100) | 27  (12 to 41) | 20  (10 to 30) |
| ≥0·3 | 0·0703  (5·86 x 10^-5^) | 78·6  (57·1 to 100) | 70  (47 to 94) | 55  (38 to 73) |
| **Total** | | | **942**  **(875 to 1028)** | **204**  **(142 to 267)** |

FPM = Fragments Per Million sequenced reads; PI = Primary Infection; CI = Confidence Interval; SD = Standard Deviation

^§^including both cfDNA-hCMV-positive samples with serology profiles fitting a recent PI and cfDNA-hCMV-positive but seronegative samples, suggestive for a very recent PI
